# Background parenchymal enhancement: behavior during neoadjuvant chemotherapy for breast cancer and relationship with a pathological complete response

**DOI:** 10.1590/0100-3984.2019.0057

**Published:** 2020

**Authors:** Sandra Regina Campos Teixeira, Hélio Sebastião Amâncio de Camargo Júnior, Cesar Cabello

**Affiliations:** 1 Centro de Atenção Integral à Saúde da Mulher da Universidade Estadual de Campinas (Caism-Unicamp), Campinas, SP, Brazil.; 2 CDE - Diagnóstico por Imagem, Campinas, SP, Brazil.

**Keywords:** Breast neoplasms/diagnostic imaging, Magnetic resonance imaging/methods, Antineoplastic agents/therapeutic use, Neoadjuvant treatment, Treatment outcome, Parenchymal tissue/diagnostic imaging, Neoplasias da mama/diagnóstico por imagem, Ressonância magnética/metodos, Antineoplásicos/uso terapêutico, Terapia neoadjuvante, Resultado do tratamento, Tecido parenquimatoso/diagnóstico por imagem

## Abstract

**Objective:**

To evaluate background parenchymal enhancement (BPE) and its characteristics, as well as its behavior before and after neoadjuvant chemotherapy (NAC), in both breasts of patients with unilateral breast cancer.

**Materials and Methods:**

This was a retrospective, cross-sectional observational study involving a consecutive sample of women with invasive breast cancer who underwent breast magnetic resonance imaging (MRI) between July 2007 and July 2017, as well as undergoing dynamic contrast-enhanced MRI before and after NAC. In both breasts, we evaluated the BPE in accordance with the Breast Imaging Reporting and Data System. We applied logistic regression analysis, and values of *p* < 0.05 were considered significant.

**Results:**

We evaluated 150 women. The mean age was 45.2 years (range, 20-74 years). The variables correlating independently with a high pre-NAC BPE, in the affected and contralateral breasts, were being under 50 years of age (odds ratio [OR] = 6.55; 95% confidence interval [95% CI]: 2.32-18.46, for both breasts) and a post-NAC BPE reduction (OR = 17.75; 95% CI: 4.94-63.73 and OR = 18.47; 95% CI: 5.19-66.49, respectively).

**Conclusion:**

Patients with invasive unilateral breast cancer who have a high pre-NAC BPE in both breasts tend to be under 50 years of age and to show a post-NAC reduction in BPE.

## Abstract (pt)

**Objetivo:**

Avaliar o realce de fundo do parênquima (RFP), suas características e seu comportamento pré- e pós-quimioterapia neoadjuvante (QTN) em ambas as mamas em pacientes com câncer de mama unilateral.

**Materiais e Métodos:**

Estudo observacional transversal retrospectivo realizado em um serviço acadêmico e um centro especializado em mamas, que incluiu mulheres com câncer de mama invasivo submetidas a QTN e que realizaram exames de ressonância magnética (RM) das mamas com contraste antes e depois da quimioterapia, entre julho de 2007 e julho de 2017. Os exames foram realizados de acordo com protocolo padronizado. O RFP foi avaliado em ambas as mamas de acordo com o ACR BI-RADS, 5ª edição. Foi aplicada análise de regressão logística. O nível de significância adotado para os testes estatísticos foi *p* < 0,05.

**Resultados:**

Foram analisadas 150 mulheres. A idade média foi de 45,2 anos (20–74 anos). Na análise de regressão multivariada, apenas a idade inferior a 50 anos e redução do RFP correlacionaram-se independentemente com o nível alto de RFP em ambas as mamas: mamas afetadas (odds ratio [OR]: 6,55; intervalo de confiança 95% [IC 95%]: 2,32–18,46 e OR: 17,75; IC 95%: 4,94–63,73, respectivamente); mamas contralaterais (OR: 6,55; IC: 95% 2,32–18,46 e OR: 18,47; IC 95%: 5,19–66,49, respectivamente).

**Conclusão:**

Idade abaixo de 50 anos e redução do RFP pós-QTN correlacionaram-se independentemente com maior RFP pré-QTN em ambas as mamas em pacientes com câncer de mama invasivo unilateral submetidas a QTN.

## INTRODUCTION

Neoadjuvant chemotherapy (NAC) has become a standard treatment for the triple-negative and human epidermal growth factor receptor 2 (HER2)-positive breast cancer subtypes^(1,2)^. Although its predicted outcomes are similar to those of adjuvant therapy, NAC has certain advantages, such as reducing the tumor burden, downstaging axillary nodal disease, and allowing early assessment of tumor response, as well as *in vivo* assessment of tumor biology^(1-4)^. It also provides some prognostic information: patients who achieve a pathological complete response (pCR) after NAC have been shown to have better survival rates^(1,2,5)^.

Dynamic contrast-enhanced magnetic resonance imaging (DCE-MRI) has been widely used in evaluating the response to NAC, allowing accurate measurement of tumor size at each stage of treatment^(4,6)^. However, it provides not only morphological information but also functional information, through the evaluation of the many factors affecting contrast uptake by the tumor and normal breast tissue^(7-9)^. One of the main parameters evaluated in DCE-MRI is background parenchymal enhancement (BPE).

**Figure 1:**
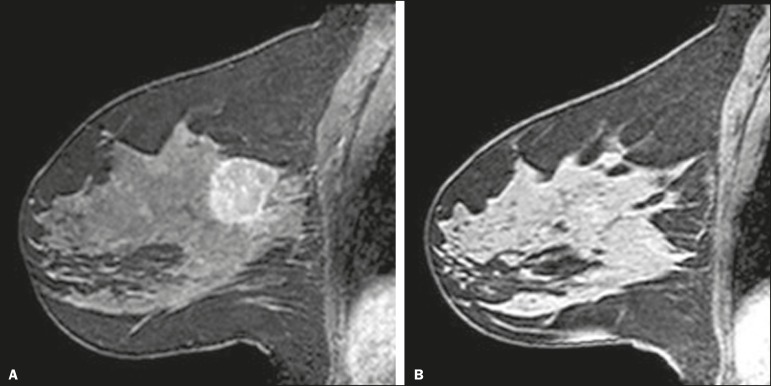
**A**: Pre-NAC contrast-enhanced sagittal DCE-MRI sequence showing a nodule with a mildly irregular form and ill-defined margins, with heterogeneous internal enhancement (invasive ductal carcinoma proven by core biopsy). **B**: Post-NAC contrast-enhanced sagittal DCE-MRI sequence not showing the previous nodule in which a pCR, according to the Response Evaluation Criteria in Solid Tumors criteria, was achieved.

The definition of BPE is enhancement of the normal fibroglandular tissue of the breast parenchyma that appears when contrast is used in MRI (Figure 1), and the BPE can be described as minimal, mild, moderate, or marked^(10)^. It is dynamic and can vary from woman to woman, as well as in a particular woman over time. It correlates with the vascularization of tissue and with its permeability to the contrast agent. Situations that alter these conditions potentially alter contrast uptake^(11)^, and several factors can influence the enhancement of breast tissue, pathological or not, focally or diffusely.

Various studies have tried to find ways to predict the response to adjuvant chemotherapy and NAC, with the purpose of enabling personalization of cancer treatment^(2,3,12,13)^. Many such studies have chosen the contralateral breast for this evaluation, in an attempt to avoid misinterpretation caused by the presence of the tumor in the affected breast. Whether the appearance of the BPE and the treatment response differ between the affected breast and the contralateral breast is an open question.

In this study, we aimed to investigate BPE in women with primary unilateral breast cancer who received NAC. To that end, we compared the BPE features, in both breasts, before and after NAC.

## MATERIALS AND METHODS

This was a retrospective, cross-sectional observational study conducted at a teaching breast care center and at a private breast imaging clinic. The study was approved by the local research ethics committee (Reference no. 2.079.528), and the patients gave written informed consent for the use of the images.

We evaluated a consecutive sample of women with invasive breast cancer who were selected to undergo NAC and who underwent DCE-MRI before and after the NAC. The study, which was conducted between January 2016 and September 2017, included women who had undergone breast MRI examinations between July 2007 and July 2017.

The inclusion criteria were having been selected to undergo NAC and the results of breast DCE-MRI performed before and after NAC being available in our radiology information system and picture archiving and communication system databases. The exclusion criteria were having a history of unilateral or bilateral mastectomy and having bilateral breast cancer.

The sample size was calculated to achieve a level of significance of 5% (α = 0.05 for a type I error) and a power of 80% (β = 0.20 for a type II error). The minimum sample size required was thus found to be n = 82.

### MRI technique

All DCE-MRI examinations of the breast were performed in a 1.5-T scanner (Achieva; Philips Medical Systems, Best, the Netherlands), with a double phased-array dedicated breast coil and the patient in the prone position. The imaging protocol included multiplanar acquisition of 0.6-mm and 4.5-mm slices in T1-weighted and T2weighted short-tau inversion-recovery, maximum intensity projection, and dynamic sequences. Bilateral sagittal T1-weighted sequences with fat saturation were acquired with the following parameters: a repetition time of 5.1 ms; an echo time of 2.3 ms; a flip angle of 10°; a 512 × 512 matrix; and a sensitivity encoding factor of 2. Images were acquired once prior to and three times after a bolus injection of 0.1 mmol/kg of gadobutrol (Dotarem; Guerbet, Roissy, France) or gadoteridol (ProHance; Bracco, Milan, Italy), which was followed by a 30 mL saline flush, digital subtraction, dynamic curve analysis, and acquisition of high-resolution isotropic sequences (in thin sections, in T1 weighting with fat saturation).

### Analysis of BPE

**Figure 2:**
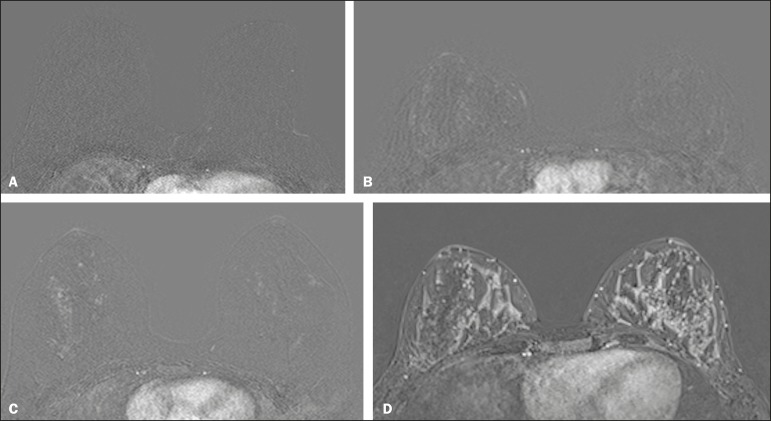
Examples of BPE levels. Subtraction sagittal sequence showing minimal BPE (**A**), mild BPE (**B**), moderate BPE (**C**), and marked BPE (**D**).

**Figure 3:**
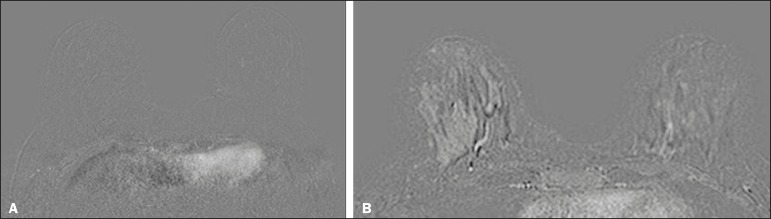
Examples of BPE symmetry. **A**: Axial subtraction showing symmetric BPE. **B**: Axial subtraction showing asymmetric BPE. The patient depicted was breastfeeding only from the right breast, with could explain the asymmetry.

**Figure 4:**
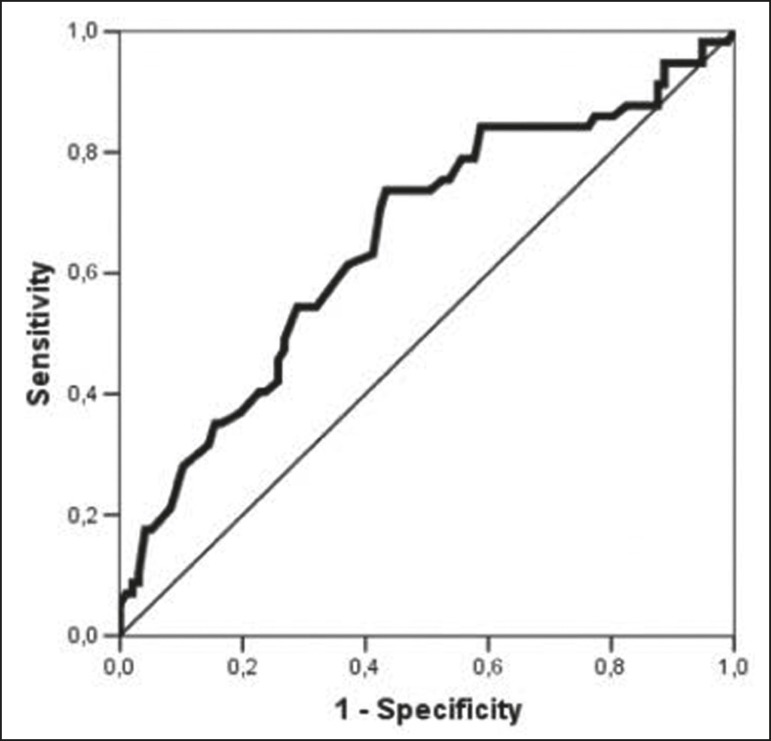
ROC curve for pCR. Area under the curve: 0.655; *p* = 0.001; 95% CI: 0.564-0.747. Cut-off point for pCR: ≤ 4.2.

We classified BPE (assessed on the first contrast-enhanced image, with fat suppression and digital subtraction, at approximately 90 s after contrast administration) in accordance with the American College of Radiology Breast Imaging Reporting and Data System (BI-RADS) Atlas, 5th edition, without quantitative calculations, as in everyday clinical practice. Both breasts were evaluated (Figure 1). Because the images were obtained in isotropic sequences, the BPE may be evaluated on sagittal and axial images, although the latter results in better evaluation of BPE symmetry. We evaluated the level and symmetry of BPE, as well as its relationship with clinical and imaging features (Figures 2 and 3). The criteria established in the St. Gallen/Vienna 2015 consensus^(14)^ were used in order to classify molecular subtypes. The pre-NAC tumor size on MRI was categorized based on a receiver operating characteristic (ROC) curve for achieving a pCR (Figure 4).

### Data analysis

The DCE-MRI data were paired with patient clinical issues, breast composition on mammography, tumor features (grade, immunohistochemical characteristics, and subtype), amount of fibroglandular tissue on MRI, features of BPE on MRI, and tumor size on MRI. The MRI assessment of both breasts included determining the amount of fibroglandular tissue, the level/symmetry of BPE, and the diameter of the tumor at its longest axis (for multicentric diseases, only the index lesion was considered).

All images were reviewed by the same radiologist, who had 10 years of experience in reading MRI scans and was blinded to all previous analyses. The results were classified and expressed as in the BI-RADS lexicon. Older examinations, performed prior to the release of the current edition of the BI-RADS, were reviewed and classified accordingly. The information about breast density on mammograms was collected directly from the database, without reassessment.

For comparison of categorical variables, we used the chi-square or Fisher’s exact test. For comparison of numerical variables, we used the Mann-Whitney and Kruskal-Wallis tests. The level of significance adopted for the statistical tests was 5% (*p* < 0.05).

## RESULTS

A total of 152 women met the criteria for inclusion. However, two were excluded because they had bilateral breast cancer. Therefore, the final sample comprised 150 women. The mean age was 45.2 years (range, 20-74 years). In each patient, both breasts presented similar results for all of the parameters analyzed.

Patients under 50 years of age presented higher pre-NAC BPE levels, in both breasts, than did those ≥ 50 years of age. Tables 1 and 2 show the demographic and clinical characteristics of the patients, together with the clinical characteristics of their affected and contralateral breasts, respectively, whereas Tables 3 and 4 show the results of the univariate and multivariate analysis of data related to the affected and contralateral breasts, respectively. In the affected breast, 53.5% of the women under 50 years of age presented moderate or marked pre-NAC BPE, compared with only 13.7% of the women ≥ 50 years of age (odds ratio [OR] = 7.24; 95% confidence interval [95% CI]: 2.97-17.64). In the contralateral breast, those proportions were 51.5% and 11.7%, respectively (OR = 7.97; 95% CI: 3.12-20.37). Premenopausal women also showed higher BPE levels. In the affected breast, 50.5% of the premenopausal women presented moderate or marked pre-NAC BPE, compared with only 18.4% of the postmenopausal women (OR = 4.53; 95% CI: 1.99-10.31). In the contralateral breast, those proportions were 47.5% and 18.4%, respectively (OR = 4.03; 95% CI: 1.77-9.16).

**Table 1:** Demographic and clinical characteristics of patients with unilateral breast cancer, together with the characteristics of the tumors in the affected breasts (n = 150).

Characteristic	Affected breast*			
	Minimal/mild (%)	Moderate/marked (%)	Total (%)	*P*
Age at dagnos s (years)				
<40	28 (31.1)	26 **(43.3)**	54 (36)	
40-49	18 (20.0)	27 **(45.0)**	45 (30)	
≥50	44 **(48.9)**	7 (11.7)	51 (34)	**<0.001**
Total	90 (100)	60 (100)	150 (100)	
Menopausal status				
Premenopausal	50 (55.6)	51 **(85.0)**	101(67.3)	
Postmenopausal	40 **(44.4)**	9 (15.0)	49 (32.7)	**<0.001**
Total	90 (100)	60 (100)	150 (100)	
Body mass index				
18.5-24.9 kg/m2 (normal-weight)	34 (47.9)	26 (56.5)	60 (513)	
25.0-29.9 kg/m2 (overweight)	26 (36.6)	14 (30.4)	40 (34.2)	
≥30.0 kg/m^2^ (Obese)	11 (15.5)	6 (13.0)	17 (14.5)	0.659
Total	71 (100)	46 (100)	117 (100)	
Tumor grade^†^				
1	3 (3.4)	1 (1-6)	4 (2.7)	
2	52 (60.2)	29 (47.5)	81 (555)	
3	32 (36.4)	29 (50.8)	61 (418)	0.359
Total	87 (100)	59 (100)	146 (100)	
HER2 status				
Negative	65 (72.2)	40 (66.7)	105 (70.0)	
Positive	25 (27.8)	20 (33.3)	45 (30.0)	0.467
Total	90 (100)	60 (100)	150 (100)	
Estrogen receptor status				
Negative	40 (44.4)	27 (45.0)	67 (44.7)	
Positive	50 (55.6)	33 (55.0)	83 (55.3)	0.947
Total	90 (100)	60 (100)	150 (100)	
Progesterone receptor status				
Negative	56 (62.2)	32 (53.3)	88 (58.7)	
Positive	34 (37.8)	28 (46.7)	62 (41.3)	0.279
Total	90 (100)	60 (100)	150 (100)	
Ki67 status				
Negative	27 (30.3)	21 (35.0)	48 (32.2)	
Positive	62 (69.7)	39 (65.0)	101 (67.8)	0.550
Total	89 (100)	60 (100)	149 (100)	
Tumor subtype				
Luminal A	14 (15.6)	12 (20.0)	26 (17.3)	
Luminal B	24 (26.7)	11 (18.3)	35 (23.3)	
HER2	12 (13.3)	9 (15.0)	21 (14.0)	
Luminal B HE32	13 (14.4)	11 (18.3)	24 (16.0)	0.772
Tripie-negative	27 (30.0)	17 (28.3)	44 (29.4)	
Total	90 (100)	60 (100)	150 (100)	
Fibroglandular tissue (on MRI)				
Almost entirely fat	0	1 (1-7)	1 (0.7)	
Scattered fibroglanoular tissue	67 (74.4)	30 (50.0)	97 (64.7)	
Heterogeneous fibroglandular tissue	22 (24.4)	25 (41.7)	47 (31.3)	**0.004**
Extreme fibroglandular tissue	1 (1.1)	4 (6.7)	5 (3.3)	
Total	90 (100)	60 (100)	150 (100)	
Breast density (on mammography)				
Almost entirely fat	3 (4.1)	0	3 (2.4)	
Scattered areas of fibroglanduar density	41 (56.2)	23 (45.1)	64 (51.6)	
Heterogeneously dense	28 (38.3)	23 (45.1)	51 (41.1)	0.051
Extremer/ dense	1 (14)	5 (9.8)	6 (4.8)	
Total	73 (100)	51 (100)	124 (100)	
Post-NAC change in BPE^‡^				
Ncre or increase	49 (54.4)	3 (5.0)	52 (34.7)	
Reduction	41 (45.6)	57 (95.0)	98 (65.3)	**<0.001**
Total	90 (100)	60 (100)	150 (100)	

* Bolding indicates significance.

† Nottingham grading system.

‡ After NAC, the BPE became symmetric in all cases and was marked in none.

**Table 2:** Demographic and clinical characteristics of patients with unilateral breast cancer, together with the characteristics of the tumors of the contralateral breasts (n = 150).

Characteristic	Contralate-al breast*			
	Minimal/mild (%)	Moderate/martted (%)	Total (%)	*P*
Age at diagnosis (years)				
<40	29 (31.2)	25 **(43.9)**	54 (36.0)	
40-49	19 (20.4)	26 **(45.6)**	45 (30.0)	
≥50	45 **(48.4)**	6 (10.5)	51 (34.0)	**< 0.001**
Total	93 (100)	57 (100)	150 (100)	
Menopausal status				
Premenopausal	53 (57.0)	48 **(84.2)**	101 (67.3)	
Postmenopausal	40 **(43.0)**	9 (15.8)	49 (32.7)	**< 0.001**
Total	93 (100)	57 (100)	150 (100)	
Body mass index				
18.5–24.9 kg/m2 (normal-weight)	38 (48.7)	24 (55.8)	60 (51.3)	
25.0-29.9 kg/m^2^ (overweight)	26 (35.1)	14 (32.6)	40 (34.2)	
≥30.0 kg/m^2^ (ocese)	12 (18.2)	5 (11.6)	17 (14.5)	0.698
Total	74 (100)	43 (100)	117 (100)	
Tumor grade^†^				
1	3 (3.3)	1 (1-6)	4 (2.7)	
2	52 (58.9)	29 (49.2)	81 (55.5)	
3	34 (37.8)	27 (49.2)	61 (41.8)	0.539
Total	69 (100)	57 (100)	146 (100)	
HER2 status				
Negative	68 (73.1)	37 (64.9)	105 (70.0)	
Positive	25 (28.9)	20 (35.1)	45 (30.0)	0.287
Total	93 (100)	57 (100)	150 (100)	
Estrogen receptor status				
Negative	40 (42.1)	27 (45.8)	67 (44.7)	
Positive	53 (57.9)	30 (54.3)	83 (55.3)	0.602
Total	93 (100)	57 (100)	150 (100)	
Progesterone receptor status				
Negative	57 (81.3)	31 (54.4)	88 (58.7)	
Positive	38 (38.7)	26 (45.6)	62 (41.3)	0.405
Total	93 (100)	57 (100)	150 (100)	
Ki67 status				
Negative	29 (31.5)	19 (33.3)	48 (32.2)	
Positive	63 (68.5)	38 (66.7)	101 (67.8)	0.818
Total	89 (100)	57 (100)	149 (100)	
Tumor subtype				
Luminal A	15 (16.1)	11 (19.3)	26 (17.3)	
Luminal B	26 (28.0)	9 (15.8)	35 (23.3)	
HER2	12 (12.9)	9 (15.8)	21 (14.0)	
Luminal BHER2	13 (14.0)	11 (19.3)	24 (16.0)	0.511
Triple-negatve	27 (29.0)	17 (29.8)	44 (29.4)	
Total	93 (100)	57 (100)	150 (100)	
Fibroglandular tissue (on MRl)				
Almost entirely fat	0	1 (1-8)	1 (0.7)	
Scattered fibroglandular tissue	68 **(73.1)**	29 (50.9)	97 (64.7)	
Heterogeneous fibroglandular tissue	24 (25.8)	23 **(40.3)**	47 (31.3)	**0.008**
Extreme fibroglandular tissue	1(1.1)	4 **(7.0)**	5 (3.3)	
Total	93 (100)	57 (100)	150 (100)	
Breast density (on mammography)				
Almost entirely fat	3 (4.0)	0	3 (2.4)	
Scattered areas of fibroglandular density	41 (54.7)	23 (46.9)	64 (51.6)	
Heterogeneously dense	30 (40.0)	21 (42.9)	51 (41.1)	0.069
Extremely dense	1 (1-3)	5 (10.2)	6 (4.8)	
Total	75 (100)	49 (100)	124 (100)	
Post-NAC charge in BPE^‡^				
None or increase	52 **(55.9)**	3 (5.3)	55 (36.7)	
Reduction	41 (44.1)	54 **(94.7)**	95 (63.3)	**< 0.001**
Total	93 (100)	57 (100)	150 (100)	

* Bolding indicates significance.

† Nottingham grading system.

‡ After NAC, the BPE became symmetric in all cases and was marked in none.

**Table 3:** Univariate and multivariate logistic regression analysis of the BPE level in the affected breasts of patients with unilateral breast cancer.

Variable	Category	Univariate analysis* (n = 150)				Stepwise multivariate analysis* (n = 137)		
		P	OR^†^	95% CI		P	OR^‡^	95% CI
Age at dagnoss (years)	≥ 50 (ref.)	-	1.00	-		-	1.00	-
	<50	**<0.001**	7.24	2.97-17.64		**<0.001**	6.88	2.45-19.34
Body mass index	18.5-24.9 kgm^2^ (normal-weight, ref.)	-	1.00	-		-	-	-
	25.0-29.9 kg/m^2^ (overweight)	0.405	0.70	0.31-1.61		-	-	-
	≥30.0 kg/m2 (obese)	0.554	0.71	0.23-2.18		-	-	-
Menopausal status	Postmenopausal (ref.)	-	1.00	-		-	-	-
	Premenopausal	**<0.001**	4.53	1.99-10.31		-	-	-
Breast density	Fat/scattered fibroglandular density (ref.)	-	1.00	-		-	-	-
	Heterogeneously/extremely dense	0.097	1.85	0.90-3.81		-	-	-
Fibroglandular tissue	Fat/scattered fibroglandular tissue (ref.)	-	1.00	-		-	-	-
	Heterogeneous/extreme	**0.005**	2.73	1.36-5.45		-	-	-
Post-NAC change in BPE, affected breast	None or increase (ref.)	—	1.00	—		—	1.00	—
	Reduction	**<0.001**	22.70	6.62-77.88		**<0.001**	17.75	4.94-63.73
Post-NAC change in BPE, contralateral breast	None or increase (ref.)	-	1.00	-		-	-	-
	Reduction	**<0.001**	10.76	4.20-27.53		-	-	-
Tumor grade^§^	1 or 2 (ref.)	-	1.00	-		-	-	-
	3	0.138	1.68	0.85-3.25		-	-	-
Tumor subtype	Luminal B (ref.)	-	1.00	-		-	-	-
	Luminal A	0.243	1.87	0.65-5.35		-	-	-
	HER2+ or Luminal B HER2+	0.238	1.75	0.69-4.40		-	-	-
	Triple-negative	0.507	1.37	0.54-3.51		-	-	-
Estrogen receptor status	Negative (ref.)	-	1.00	-		-	-	-
	Positive	0.947	0.98	0.51-1.89		-	-	-
Progesterone receptor status	Negatve (ref.)	-	1.00	-		-	-	-
	Positive	0.280	144	0.74-2.80		-	-	-
HER2 status	Negative (ref.)	-	1.00	-		-	-	-
	Positive	0.468	1.30	0.64-2.64		-	-	-
Ki67 status	Negative (ref.)	—	1.00	—		—	-	—
	Positive	0.551	0.81	0.40-1.62		-	-	-
Clinical pre-NAC tumor size	Continuous variable	0.703	0.964	0.798-1.165		-	-	-
Ultrasound pre-NAC tumor size	Continuous variable	0.183	0.860	0.690-1.073		-	-	-
MRI pre-NAC tumor size	Continuous variable	0.598	0.9S8	0.816-1.124		-	-	-

ref., reference.

* Bolding indicates significance.

† For moderate/marked pre-NAC BPE (n = 56) vs. minimal/mild pre-NAC BPE (n = 81).

‡ For moderate/marked pre-NAC BPE (n = 60) vs. minimal/mild pre-NAC BPE (n = 90).

§ Nottingham grading system.

**Table 4:** Univariate and multivariate logistic regression analysis of the BPE level in the contralateral breasts of patients with unilateral breast cancer.

Variable	Category	Univariate analysis* (n = 150)				Stepwise multivariate analysis* (n = 137)		
		P	OR^†^	95% CI		P	OR^‡^	95% CI
Age at diagnosis (years)	≥ 50 (ref.)	—	1.00	—		—	1.00	—
	<50	**<0.001**	7.97	3.12-20.37		**<0.001**	6.55	2.32-18.46
Body mass index	18.5-24.9 kg/m2 (normal-weight, ref.)	-	1.00	-		-	-	-
	25.0-29.9 kg/m^2^ (overweight)	0.614	0.81	0.35-1.85		-	-	-
	≥ 30.0 kg'm^2^ (obese)	0.429	0.63	0.20-2.00		-	-	-
Menopausal status	Postmenopausal (ref.)	-	1.00	-		-	-	-
	Premenopausal	**<0.001**	4.03	1.77-9.16		-	-	-
Breast density	Fat/scattered fibroglandular density (ref.)	-	1.00	-		-	-	-
	Heterogeneously/extremely dense	0.201	1.60	0.78-3.31		-	-	-
Fibroglandular tissue	Fat/scattered fibroglandular tissue (ref.)	-	1.00	-		-	-	-
	Heterogeneous/extreme	**0.011**	2.45	1.22-4.90		-	-	-
Post-NAC change in BPE, affected breast	None or increase (ref.)	-	1.00	-		-	-	-
	Reduction	**<0.001**	20.04	585-68.66		-	-	-
Post-NAC change in BPE, contralateral breast	None or increase (ref.)	-	1.00	-		-	1.00	-
	Reduction	**<0.001**	22.83	6.66-78.30		**<0.001**	18.57	5.19-68.49
Tumor grade^§^	1 or 2 (ref.)	-	1.00	-		-	-	-
	3	0.274	1.46	0.74-2.85		-	-	-
Tumor subtype	Luminal B (ref.)	-	1.00	-		-	-	-
	Luminal A	0.176	2.12	0.72-628		-	-	-
	HER2+ or Luminal B HER2+	0.087	2.31	0.89-6.03		-	-	-
	Triple-negative	0.227	1.82	0.69-4.80		-	-	-
Estrogen receptor status	Negative (ref.)	-	1.00	-		-	-	-
	Positive	0.603	0.84	0.43-L63		-	-	-
Progesterone receptor status	Negative (ref.)	-	1.00	-		-	-	-
	Positive	0.405	1.33	0.68-2.59		-	-	-
HER2 status	Negative (ref.)	-	1.00	-		-	-	-
	Positive	0.288	1.47	0.72-3.00		-	-	-
Ki67 status	Negative (ref.)	-	1.00	-		-	-	-
	Positive	0.818	0.92	0.46-1.86		-	-	-
Clinical pre-NAC tumor size	Continuous variable	0.420	0.923	0.759-1.22		-	-	-
Ultrasound pre-NAC tumor size	Continuous variable	0.287	0.887	0.711-1.106		-	-	-
MRI pre-NAC tumor size	Continuous variable	0.267	0.911	0.773-1.074		—	—	—

ref., reference.

* Bolding indicates significance.

† For moderate/marked pre-NAC BPE (n = 56) vs. minimal/mild pre-NAC BPE (n = 81).

‡ For moderate/marked pre-NAC BPE (n = 60) vs. minimal/mild pre-NAC BPE (n = 90).

§ Nottingham grading system.

The univariate analysis showed that, for the affected breasts, 25.5% of the women who had low pre-NAC BPE levels presented heterogeneous or extreme fibroglandular tissue, whereas 48.4% of those who had high pre-NAC BPE levels presented such tissue (OR = 2.73; 95% CI: 1.36-5.45), as can be seen in Tables 1 and 3. For the contralateral breasts, 26.9% of the women who had low pre-NAC BPE levels presented heterogeneous or extreme fibroglandular tissue, whereas 47.3% of those who had high pre-NAC BPE levels presented these patterns (OR = 2.45; 95% CI: 1.22-4.90), as can be seen in Tables 2 and 4. The proportion of affected breasts showing a post-NAC reduction in BPE was 95.0% for those presenting high pre-NAC BPE levels and 45.6% for those presenting low pre-NAC BPE levels (OR = 22.7; 95% CI: 6.62-77.86). For the contralateral breasts, those proportions were 94.7% and 44.1%, respectively (OR = 20.04; 95% CI: 5.85-68.66).

In the multivariate regression analysis, only being under 50 years of age and a post-NAC reduction in BPE were independently correlated with a high pre-NAC BPE level in both breasts. In the affected breasts (Table 3), being under 50 years of age showed an OR of 6.55 (95% CI: 2.32-18.46) and a post-NAC reduction in BPE showed an OR of 17.75 (95% CI: 4.94-63.73).

In the contralateral breasts (Table 4), being under 50 years of age showed an OR of 6.55 (95% CI: 2.32-18.46) and a post-NAC reduction in BPE showed an OR of 18.47 (95% CI: 5.19-66.49). The pre-NAC BPE level was not found to correlate with body mass index, breast density, tumor grade, estrogen receptor status, progesterone receptor status, HER2 status, Ki67 status, or tumor subtype. When adjusted for the pre-NAC tumor size on MRI, luminal subtype, age, tumor grade, and menopausal status, the multivariate analysis also showed that a pre-NAC reduction in BPE, in the affected or contralateral breast, was an independent predictor of achieving a pCR (Table 5).

**Table 5:** Multivariate logistic regression of factors associated with achieving a pCR, adjusted for pre-NAC tumor size on MRI, luminal subtype, patient age, tumor grade, and menopausal status in patients with unilateral breast cancer (n = 146).

variable	Category	P*	OR^†^	95% Cl
Symmetry of pre-NAC BPE	Asymmetric (reference)	-	1.00	-
	Symmetric	0.316	4.21	0.11-15L98
Level of pre-NAC BPE, affected breast	Minimal (reference)	-	1.00	-
	Mild	0.728	1.19	0.44-3.22
	Moderate	0.375	1.66	0.54-5.04
	Marked	0.829	0.87	0.24-3.13
Level of pre-NAC BPE, contralateral breast	Minimal (reference)	-	1.00	-
	Mild	0.811	1.13	0.42-3.04
	Moderate	0.302	1.80	0.59-5.53
	Marked	0.919	0.94	0.26-3.41
Post-NAC change in BPE, affected breast	None or increase (reference)	-	1.00	-
	Reduction	**0.007**	2.94	1.34 - 6.42
Post-NAC change in BPE, contralateral breast	None or increase (reference)	-	1.00	-
	Reduction	**0.003**	3.19	1.48-6.91

* Bolding indicates significance.

† For achieving a pCR (n = 67) vs. not achieving a pCR (n = 79).

## DISCUSSION

In addition to tumor enhancement on breast MRI, which has been shown to be an important marker of a response to NAC, another component, BPE, also appears to be a useful parameter in patients with unilateral breast cancer undergoing NAC. Müller-Schimpfle et al.^(15)^ studied BPE variation during the menstrual cycle and its relationship with age. The authors found that the level of BPE was higher in patients between 35 and 50 years of age than among those who were younger or older. They also showed that postmenopausal women typically have a lower level of BPE than do premenopausal women.

In the present study, being under age 50 and having a post-NAC reduction in BPE were independently correlated with a higher pre-NAC BPE level, in both breasts. Both of those factors are clearly correlated with hormonal status, as has previously been reported^(11,15-19)^. Therefore, we can extrapolate a correlation between hormonal status and higher BPE. This suggests that the breasts of premenopausal women have greater vascularization, greater permeability to the contrast agent, or both.

Schrading et al.^(20)^studied the influence of taxanes on the response assessment with DCE-MRI and showed that even the normal (contralateral) breasts present modifications after NAC. The ovarian suppression caused by chemotherapy could explain their findings, at least in part.

Chen et al.^(8)^ also found that higher BPE before NAC in contralateral breasts was associated with a greater reduction in BPE after the treatment. It should be borne in mind that all of this behavior occurs bilaterally. Although this might have been supposed, confirmatory data were not yet available, because all previous studies of the topic involved only women without cancer or only the contralateral breast of women with unilateral invasive cancer^(7,8,20-23)^. Our findings allow us to conclude that the behavior of BPE is unaffected by the presence of the tumor in the affected breast or by the absence of a tumor in the contralateral breast. The lack of data from affected breasts is notable, because of the possibility of misinterpretation between parenchymal and tumor enhancement. The similarity presented in our study supports a variety of translational possibilities.

Diffuse BPE, if it is moderate or marked, may interfere with the accuracy of DCE-MRI, reducing its capacity to detect small lesions^(19,24)^. In the present study, neither breast density nor the histopathological features of the tumor correlated with BPE levels, supporting the idea that BPE could be an imaging biomarker of a response to NAC that is independent of tumor features.

When the BPE is asymmetrical, it may be confused with non-mass enhancement, mostly when it appears as regional or focal. Baltzer et al.^(25)^ found that lesions with non-mass-like enhancement were the main cause of falsepositive findings leading to biopsy. Only four of the 150 women in our sample presented with diffuse asymmetric BPE, which became symmetric after NAC in all four.

A number of studies have shown that BPE changes throughout the menstrual cycle, being less intense and more diffuse in the second week^(15,16,19)^. Because of the urgency of the cases in our sample, the stage of the menstrual cycle was not taken into account in the scheduling of the examinations, which constitutes a limitation of our study.

It is noteworthy that we found considerable similarity between the affected and contralateral breasts. Contrary to previous suppositions, our data show that the presence of a tumor does not alter BPE. That allows BPE to be used as a marker of a response to NAC, even in cases of bilateral breast cancer or contralateral mastectomy.
